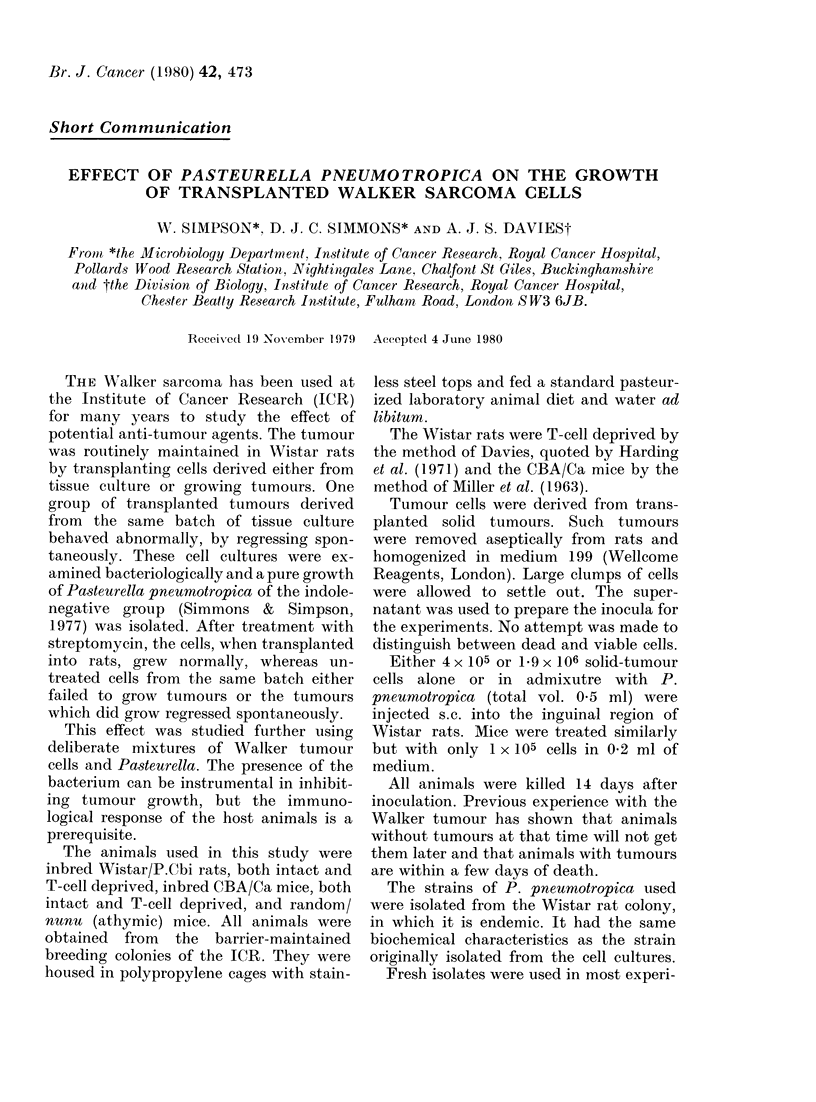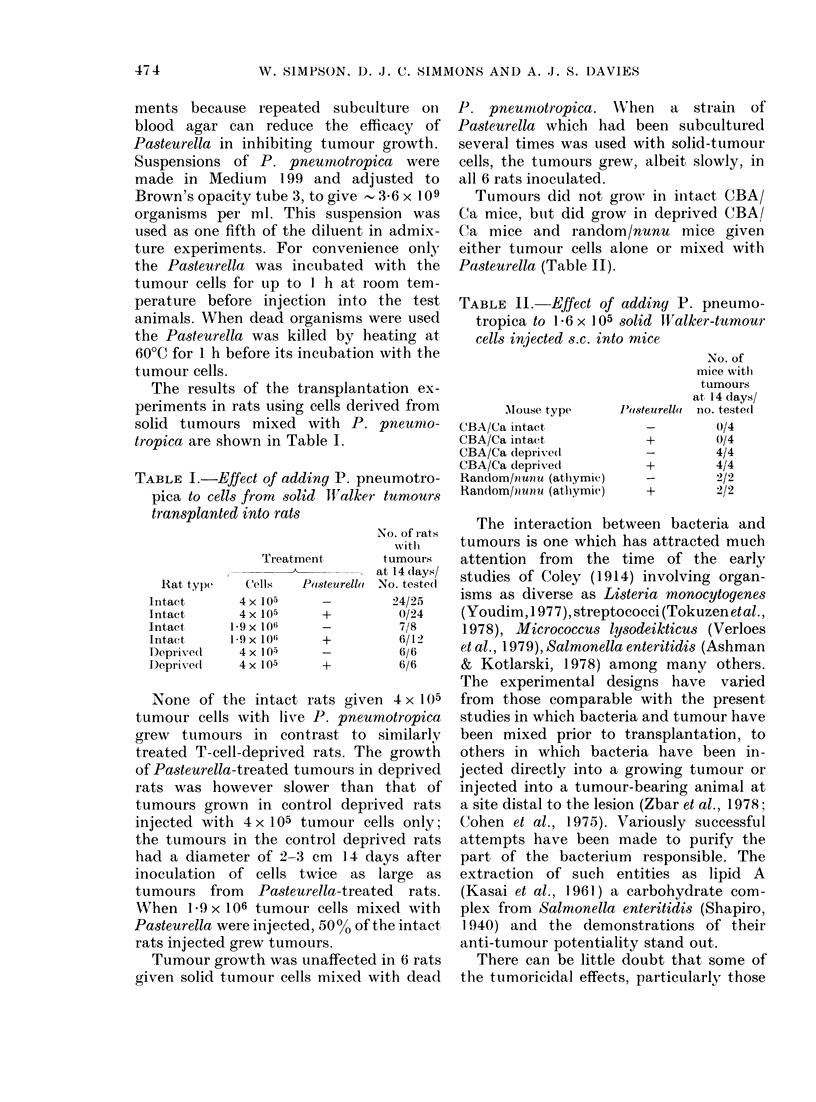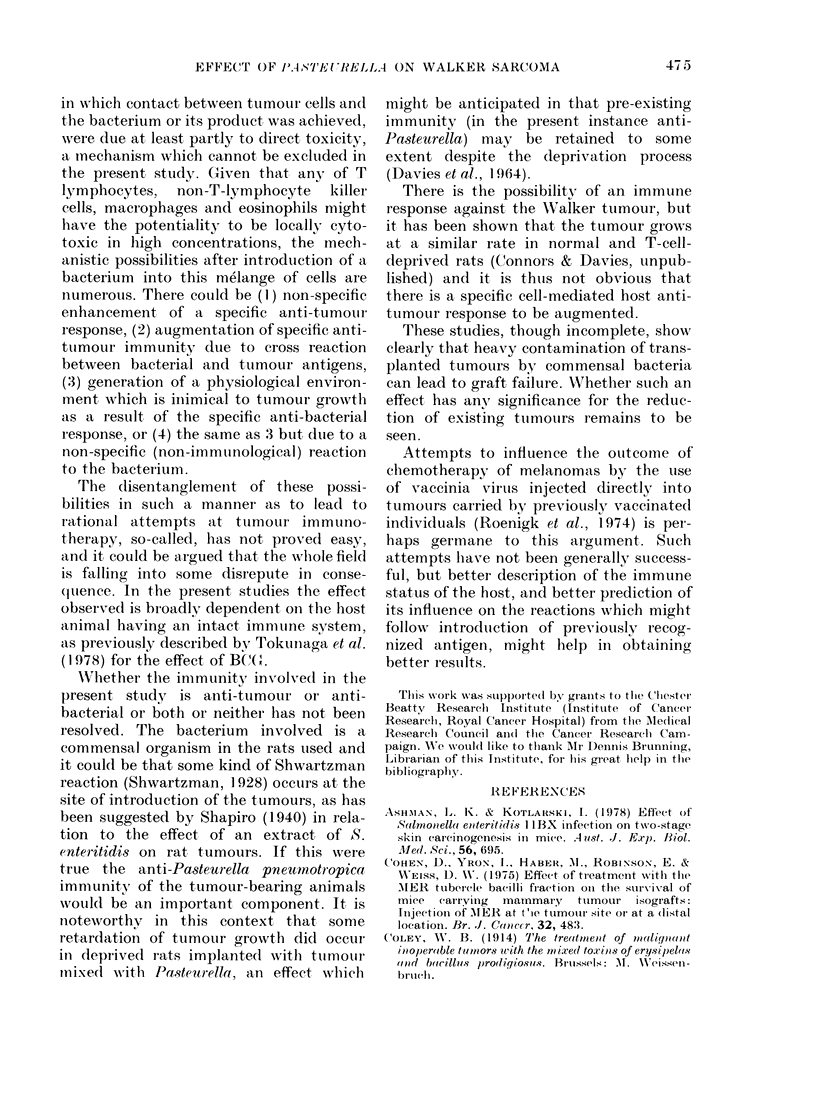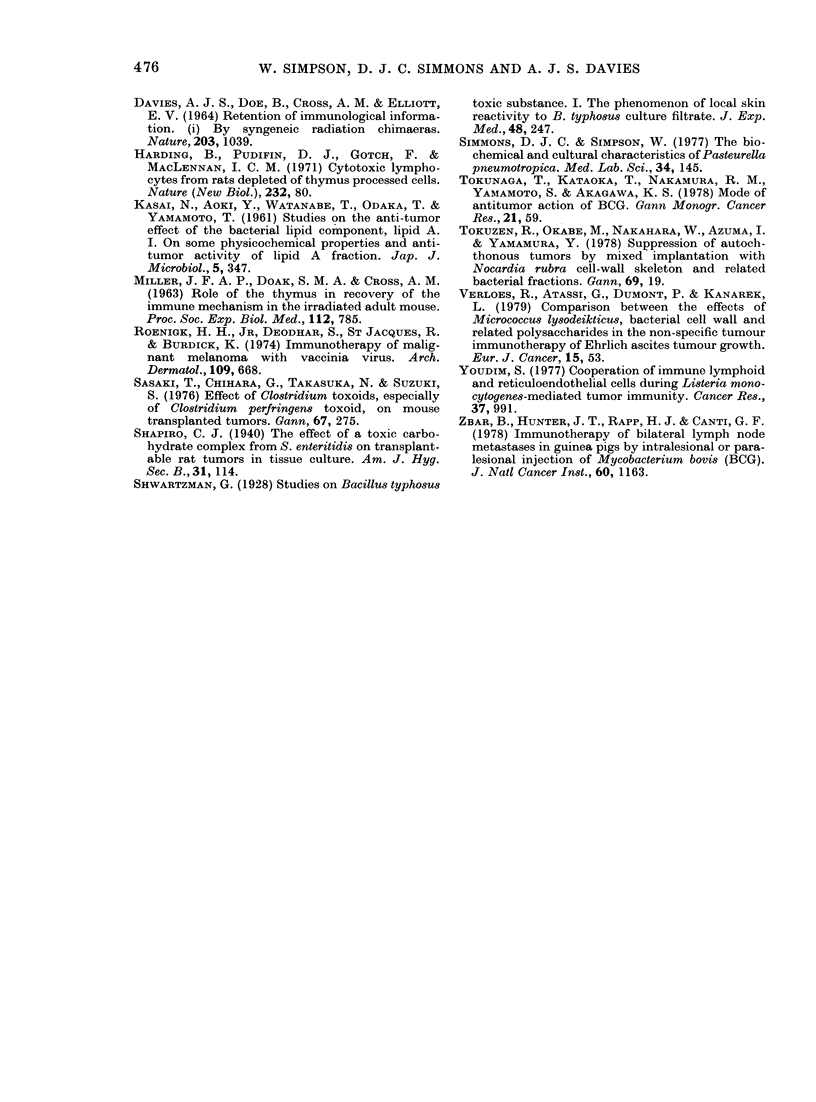# Effect of Pasteurella pneumotropica on the growth of transplanted Walker sarcoma cells.

**DOI:** 10.1038/bjc.1980.260

**Published:** 1980-09

**Authors:** W. Simpson, D. J. Simmons, A. J. Davies


*Br. J. Cancer* (1980) **42**, 473

***Short Communication***

### EFFECT OF *PASTEURELLA PNEUMOTROPICA* ON THE GROWTH OF TRANSPLANTED WALKER SARCOMA CELLS

W. SIMPSON*, D. J. C. SIMMONS* AND A. J. S. DAVIES†

*From *the Microbiology Department, Institute of Cancer Research, Royal Cancer Hospital, Pollards Wood Research Station, Nightingales Lane, Chalfont St Giles, Buckinghamshire and †the Division of Biology, Institute of Cancer Research, Royal Cancer Hospital, Chester Beatty Research Institute, Fulham Road, London SW3 6JB.*

Received 19 November 1979 Accepted 4 June 1980

THE Walker sarcoma has been used at the Institute of Cancer Research (ICR) for many years to study the effect of potential anti-tumour agents. The tumour was routinely maintained in Wistar rats by transplanting cells derived either from tissue culture or growing tumours. One group of transplanted tumours derived from the same batch of tissue culture behaved abnormally, by regressing spontaneously. These cell cultures were examined bacteriologically and a pure growth of *Pasteurella pneumotropica* of the indole-negative group (Simmons & Simpson, 1977) was isolated. After treatment with streptomycin, the cells, when transplanted into rats, grew normally, whereas untreated cells from the same batch either failed to grow tumours or the tumours which did grow regressed spontaneously.

This effect was studied further using deliberate mixtures of Walker tumour cells and *Pasteurella*. The presence of the bacterium can be instrumental in inhibiting tumour growth, but the immunological response of the host animals is a prerequisite.

The animals used in this study were inbred Wistar/P.Cbi rats, both intact and T-cell deprived, inbred CBA/Ca mice, both intact and T-cell deprived, and random/*nunu* (athymic) mice. All animals were obtained from the barrier-maintained breeding colonies of the ICR. They were housed in polypropylene cages with stainless steel tops and fed a standard pasteurized laboratory animal diet and water *ad libitum*.

The Wistar rats were T-cell deprived by the method of Davies, quoted by Harding *et al.* (1971) and the CBA/Ca mice by the method of Miller *et al.* (1963).

Tumour cells were derived from transplanted solid tumours. Such tumours were removed aseptically from rats and homogenized in medium 199 (Wellcome Reagents, London). Large clumps of cells were allowed to settle out. The supernatant was used to prepare the inocula for the experiments. No attempt was made to distinguish between dead and viable cells.

Either $4 \times 10^5$ or $1{\cdot}9 \times 10^6$ solid-tumour cells alone or in admixutre with *P. pneumotropica* (total vol. 0·5 ml) were injected s.c. into the inguinal region of Wistar rats. Mice were treated similarly but with only $1 \times 10^5$ cells in 0·2 ml of medium.

All animals were killed 14 days after inoculation. Previous experience with the Walker tumour has shown that animals without tumours at that time will not get them later and that animals with tumours are within a few days of death.

The strains of *P. pneumotropica* used were isolated from the Wistar rat colony, in which it is endemic. It had the same biochemical characteristics as the strain originally isolated from the cell cultures.

Fresh isolates were used in most experiments because repeated subculture on blood agar can reduce the efficacy of *Pasteurella* in inhibiting tumour growth. Suspensions of *P. pneumotropica* were made in Medium 199 and adjusted to Brown's opacity tube 3, to give $\sim 3{\cdot}6 \times 10^9$ organisms per ml. This suspension was used as one fifth of the diluent in admixture experiments. For convenience only the *Pasteurella* was incubated with the tumour cells for up to 1 h at room temperature before injection into the test animals. When dead organisms were used the *Pasteurella* was killed by heating at 60°C for 1 h before its incubation with the tumour cells.

The results of the transplantation experiments in rats using cells derived from solid tumours mixed with *P. pneumotropica* are shown in Table I.

TABLE I.—*Effect of adding* P. pneumotropica *to cells from solid Walker tumours transplanted into rats*

| Rat type | Treatment: Cells | Treatment: *Pasteurella* | No. of rats with tumours at 14 days/ No. tested |
|---|---|---|---|
| Intact | $4 \times 10^5$ | – | 24/25 |
| Intact | $4 \times 10^5$ | + | 0/24 |
| Intact | $1{\cdot}9 \times 10^6$ | – | 7/8 |
| Intact | $1{\cdot}9 \times 10^6$ | + | 6/12 |
| Deprived | $4 \times 10^5$ | – | 6/6 |
| Deprived | $4 \times 10^5$ | + | 6/6 |

None of the intact rats given $4 \times 10^5$ tumour cells with live *P. pneumotropica* grew tumours in contrast to similarly treated T-cell-deprived rats. The growth of *Pasteurella*-treated tumours in deprived rats was however slower than that of tumours grown in control deprived rats injected with $4 \times 10^5$ tumour cells only; the tumours in the control deprived rats had a diameter of 2–3 cm 14 days after inoculation of cells twice as large as tumours from *Pasteurella*-treated rats. When $1{\cdot}9 \times 10^6$ tumour cells mixed with *Pasteurella* were injected, 50% of the intact rats injected grew tumours.

Tumour growth was unaffected in 6 rats given solid tumour cells mixed with dead *P. pneumotropica*. When a strain of *Pasteurella* which had been subcultured several times was used with solid-tumour cells, the tumours grew, albeit slowly, in all 6 rats inoculated.

Tumours did not grow in intact CBA/Ca mice, but did grow in deprived CBA/Ca mice and random/*nunu* mice given either tumour cells alone or mixed with *Pasteurella* (Table II).

TABLE II.—*Effect of adding* P. pneumotropica *to* $1{\cdot}6 \times 10^5$ *solid Walker-tumour cells injected s.c. into mice*

| Mouse type | *Pasteurella* | No. of mice with tumours at 14 days/ no. tested |
|---|---|---|
| CBA/Ca intact | – | 0/4 |
| CBA/Ca intact | + | 0/4 |
| CBA/Ca deprived | – | 4/4 |
| CBA/Ca deprived | + | 4/4 |
| Random/*nunu* (athymic) | – | 2/2 |
| Random/*nunu* (athymic) | + | 2/2 |

The interaction between bacteria and tumours is one which has attracted much attention from the time of the early studies of Coley (1914) involving organisms as diverse as *Listeria monocytogenes* (Youdim, 1977), streptococci (Tokuzen *et al.*, 1978), *Micrococcus lysodeikticus* (Verloes *et al.*, 1979), *Salmonella enteritidis* (Ashman & Kotlarski, 1978) among many others. The experimental designs have varied from those comparable with the present studies in which bacteria and tumour have been mixed prior to transplantation, to others in which bacteria have been injected directly into a growing tumour or injected into a tumour-bearing animal at a site distal to the lesion (Zbar *et al.*, 1978; Cohen *et al.*, 1975). Variously successful attempts have been made to purify the part of the bacterium responsible. The extraction of such entities as lipid A (Kasai *et al.*, 1961) a carbohydrate complex from *Salmonella enteritidis* (Shapiro, 1940) and the demonstrations of their anti-tumour potentiality stand out.

There can be little doubt that some of the tumoricidal effects, particularly those in which contact between tumour cells and the bacterium or its product was achieved, were due at least partly to direct toxicity, a mechanism which cannot be excluded in the present study. Given that any of T lymphocytes, non-T-lymphocyte killer cells, macrophages and eosinophils might have the potentiality to be locally cytotoxic in high concentrations, the mechanistic possibilities after introduction of a bacterium into this mélange of cells are numerous. There could be (1) non-specific enhancement of a specific anti-tumour response, (2) augmentation of specific anti-tumour immunity due to cross reaction between bacterial and tumour antigens, (3) generation of a physiological environment which is inimical to tumour growth as a result of the specific anti-bacterial response, or (4) the same as 3 but due to a non-specific (non-immunological) reaction to the bacterium.

The disentanglement of these possibilities in such a manner as to lead to rational attempts at tumour immunotherapy, so-called, has not proved easy, and it could be argued that the whole field is falling into some disrepute in consequence. In the present studies the effect observed is broadly dependent on the host animal having an intact immune system, as previously described by Tokunaga *et al.* (1978) for the effect of BCG.

Whether the immunity involved in the present study is anti-tumour or anti-bacterial or both or neither has not been resolved. The bacterium involved is a commensal organism in the rats used and it could be that some kind of Shwartzman reaction (Shwartzman, 1928) occurs at the site of introduction of the tumours, as has been suggested by Shapiro (1940) in relation to the effect of an extract of *S. enteritidis* on rat tumours. If this were true the anti-*Pasteurella pneumotropica* immunity of the tumour-bearing animals would be an important component. It is noteworthy in this context that some retardation of tumour growth did occur in deprived rats implanted with tumour mixed with *Pasteurella*, an effect which might be anticipated in that pre-existing immunity (in the present instance anti-*Pasteurella*) may be retained to some extent despite the deprivation process (Davies *et al.*, 1964).

There is the possibility of an immune response against the Walker tumour, but it has been shown that the tumour grows at a similar rate in normal and T-cell-deprived rats (Connors & Davies, unpublished) and it is thus not obvious that there is a specific cell-mediated host anti-tumour response to be augmented.

These studies, though incomplete, show clearly that heavy contamination of transplanted tumours by commensal bacteria can lead to graft failure. Whether such an effect has any significance for the reduction of existing tumours remains to be seen.

Attempts to influence the outcome of chemotherapy of melanomas by the use of vaccinia virus injected directly into tumours carried by previously vaccinated individuals (Roenigk *et al.*, 1974) is perhaps germane to this argument. Such attempts have not been generally successful, but better description of the immune status of the host, and better prediction of its influence on the reactions which might follow introduction of previously recognized antigen, might help in obtaining better results.

This work was supported by grants to the Chester Beatty Research Institute (Institute of Cancer Research, Royal Cancer Hospital) from the Medical Research Council and the Cancer Research Campaign. We would like to thank Mr Dennis Brunning, Librarian of this Institute, for his great help in the bibliography.

### REFERENCES

ASHMAN, L. K. & KOTLARSKI, I. (1978) Effect of *Salmonella enteritidis* 11BX infection on two-stage skin carcinogenesis in mice. *Aust. J. Exp. Biol. Med. Sci.*, **56**, 695.

COHEN, D., YRON, I., HABER, M., ROBINSON, E. & WEISS, D. W. (1975) Effect of treatment with the MER tubercle bacilli fraction on the survival of mice carrying mammary tumour isografts: Injection of MER at the tumour site or at a distal location. *Br. J. Cancer*, **32**, 483.

COLEY, W. B. (1914) *The treatment of malignant inoperable tumors with the mixed toxins of erysipelas and bacillus prodigiosus.* Brussels: M. Weissenbruch.

DAVIES, A. J. S., DOE, B., CROSS, A. M. & ELLIOTT, E. V. (1964) Retention of immunological information. (i) By syngeneic radiation chimaeras. *Nature*, **203**, 1039.

HARDING, B., PUDIFIN, D. J., GOTCH, F. & MACLENNAN, I. C. M. (1971) Cytotoxic lymphocytes from rats depleted of thymus processed cells. *Nature* (*New Biol.*), **232**, 80.

KASAI, N., AOKI, Y., WATANABE, T., ODAKA, T. & YAMAMOTO, T. (1961) Studies on the anti-tumor effect of the bacterial lipid component, lipid A. I. On some physicochemical properties and antitumor activity of lipid A fraction. *Jap. J. Microbiol.*, **5**, 347.

MILLER, J. F. A. P., DOAK, S. M. A. & CROSS, A. M. (1963) Role of the thymus in recovery of the immune mechanism in the irradiated adult mouse. *Proc. Soc. Exp. Biol. Med.*, **112**, 785.

ROENIGK, H. H., JR, DEODHAR, S., ST JACQUES, R. & BURDICK, K. (1974) Immunotherapy of malignant melanoma with vaccinia virus. *Arch. Dermatol.*, **109**, 668.

SASAKI, T., CHIHARA, G., TAKASUKA, N. & SUZUKI, S. (1976) Effect of *Clostridium* toxoids, especially of *Clostridium perfringens* toxoid, on mouse transplanted tumors. *Gann*, **67**, 275.

SHAPIRO, C. J. (1940) The effect of a toxic carbohydrate complex from *S. enteritidis* on transplantable rat tumors in tissue culture. *Am. J. Hyg. Sec. B.*, **31**, 114.

SHWARTZMAN, G. (1928) Studies on *Bacillus typhosus* toxic substance. I. The phenomenon of local skin reactivity to *B. typhosus* culture filtrate. *J. Exp. Med.*, **48**, 247.

SIMMONS, D. J. C. & SIMPSON, W. (1977) The biochemical and cultural characteristics of *Pasteurella pneumotropica*. *Med. Lab. Sci.*, **34**, 145.

TOKUNAGA, T., KATAOKA, T., NAKAMURA, R. M., YAMAMOTO, S. & AKAGAWA, K. S. (1978) Mode of antitumor action of BCG. *Gann Monogr. Cancer Res.*, **21**, 59.

TOKUZEN, R., OKABE, M., NAKAHARA, W., AZUMA, I. & YAMAMURA, Y. (1978) Suppression of autochthonous tumors by mixed implantation with *Nocardia rubra* cell-wall skeleton and related bacterial fractions. *Gann*, **69**, 19.

VERLOES, R., ATASSI, G., DUMONT, P. & KANAREK, L. (1979) Comparison between the effects of *Micrococcus lysodeikticus*, bacterial cell wall and related polysaccharides in the non-specific tumour immunotherapy of Ehrlich ascites tumour growth. *Eur. J. Cancer*, **15**, 53.

YOUDIM, S. (1977) Cooperation of immune lymphoid and reticuloendothelial cells during *Listeria monocytogenes*-mediated tumor immunity. *Cancer Res.*, **37**, 991.

ZBAR, B., HUNTER, J. T., RAPP, H. J. & CANTI, G. F. (1978) Immunotherapy of bilateral lymph node metastases in guinea pigs by intralesional or paralesional injection of *Mycobacterium bovis* (BCG). *J. Natl Cancer Inst.*, **60**, 1163.